# Ignore Similarity If You Can: A Computational Exploration of Exemplar Similarity Effects on Rule Application

**DOI:** 10.3389/fpsyg.2017.00424

**Published:** 2017-03-21

**Authors:** Duncan P. Brumby, Ulrike Hahn

**Affiliations:** ^1^UCL Interaction Centre, University College LondonLondon, UK; ^2^Department of Psychological Sciences, Birkbeck, University of LondonLondon, UK

**Keywords:** categorization, rules, similarity, computational model, hybrid models

## Abstract

It is generally assumed that when making categorization judgments the cognitive system learns to focus on stimuli features that are relevant for making an accurate judgment. This is a key feature of hybrid categorization systems, which selectively weight the use of exemplar- and rule-based processes. In contrast, Hahn et al. (2010) have shown that people cannot help but pay attention to exemplar similarity, even when doing so leads to classification errors. This paper tests, through a series of computer simulations, whether a hybrid categorization model developed in the ACT-R cognitive architecture (by Anderson and Betz, 2001) can account for the Hahn et al. dataset. This model implements Nosofsky and Palmeri’s (1997) exemplar-based random walk model as its exemplar route, and combines it with an implementation of Nosofsky et al. (1994) rule-based model RULEX. A thorough search of the model’s parameter space showed that while the presence of an exemplar-similarity effect on response times was associated with classification errors it was possible to fit both measures to the observed data for an unsupervised version of the task (i.e., in which no feedback on accuracy was given). Difficulties arose when the model was applied to a supervised version of the task in which explicit feedback on accuracy was given. Modeling results show that the exemplar-similarity effect is diminished by feedback as the model learns to avoid the error-prone exemplar-route, taking instead the accurate rule-route. In contrast to the model, Hahn et al. found that people continue to exhibit robust exemplar-similarity effects even when given feedback. This work highlights a challenge for understanding how and why people combine rules and exemplars when making categorization decisions.

## Introduction

A classic distinction in the categorization literature is between categorization by rule as opposed to categorization by exemplar similarity (Nosofsky et al., 1994; Nosofsky and Palmeri, 1997). More recently, these two alternatives have been fused in hybrid systems of the categorization process. Hybrid models combine rule and exemplar processing within a single framework (e.g., Vandierendonck, 1995; Palmeri, 1997; Ashby et al., 1998; Erickson and Kruschke, 1998; Anderson and Betz, 2001). These models vary in the way rules and exemplars are related. For example, rule- and exemplar-routes might be subject to global or local, trial-by-trial competition, or the outputs of each route might be blended into a composite response. A core assumption of virtually all these hybrid models, however, is that the relative influence of rule and exemplar processes is influenced by their *utility*. In other words, sensitivity to exemplar-similarity is subject to strategic control and the system learns to focus on stimuli dimensions that lead to accurate classification judgments.

In contrast to this prevailing theoretical view, Hahn et al. (2010) have shown that people cannot help but pay attention to exemplar similarity, even when doing so leads to classification error (see also, von Helversen et al., 2013). Across a series of experiments, participants were explicitly provided with a perfectly predictive classification rule along with a series of positive examples to illustrate the rule. After viewing the training items, participants were asked to classify novel test items that either complied with or violated the rule. Half of the items had high similarity to the training items, and half had low similarity to the training items. In contrast to previous work on similarity and explicit rule use (e.g., Allen and Brooks, 1991; Regehr and Brooks, 1995; Lacroix et al., 2005; Folstein and van Petten, 2010; but see also Folstein et al., 2008), the similarity manipulation depended entirely on features that were not mentioned in the rule and which were consequently entirely irrelevant to correct classification. Furthermore, given the design of the test set, responding based on similarity alone would lead to chance performance (i.e., accuracy levels of 50%).

Even though the similarity manipulation in Hahn et al.’s (2010) study was orthogonal to category membership, robust effects of exemplar similarity were observed. Specifically, participants’ correct responses to rule-compliant test items that were also similar to the items viewed at training were faster than those to equally compliant items that were not. These effects could not be explained by a simple failure of participants to apply the given rule because participants made very few categorization errors (mean error-rates were between 5 and 9%). In other words, participants were sensitive to exemplar-similarity effects while making very few classification errors, and these similarity effects persisted over the course of the experiment (see Hahn et al., section 6). This opens up important questions about how rules and exemplars were being combined when making categorization decisions in Hahn et al.’s rule application task.

In this paper, we conduct a detailed computational exploration of Anderson and Betz’s (2001) hybrid model of categorization by applying it to Hahn et al.’s (2010) rule application task. Anderson and Betz’s model implements two well-known models of categorization, Nosofsky and Palmeri’s (1997) exemplar based random walk model (EBRW) and Nosofsky et al. (1994) rule-plus-exception (RULEX) model, within the general cognitive architecture of ACT-R (Anderson et al., 2004). As a result, it provides a computationally explicit means for capturing the interaction between exemplar-based and rule-based classification within a single system. It is comprehensive and explicitly defined in all aspects of the task, from feature uptake through to production of a response. The model also provides quantitative predictions of both errors and response times, and, in this regard, remains (to the best of our knowledge) the only hybrid rule-exemplar model in the literature to do so. Finally, it has been validated (see Anderson and Betz, 2001) in its match to both EBRW and RULEX predictions, as well as in its ability to capture data indicative of rule-exemplar interaction from the studies of Erickson and Kruschke (1998).

Given this, we consider whether the model can be made to fit the observed data from Hahn et al.’s (2010) studies, both in terms of response time and error-rate. This is not a forgone conclusion because, as we shall explain below, the use of exemplar-based processes tends to result in response errors. We therefore use the model to systematically explore the response patterns produced by various blends of rule-based and exemplar-based processes and whether a fit to data can be achieved.

In the following, we first describe our implementation of Anderson and Betz’s (2001) model in the current version of the ACT-R software (ACT-R 6, Anderson et al., 2004) and then evaluate the model against data from Hahn et al.’s (2010) rule application task. We end by discussing the implications of the results of this modeling study for hybrid categorization models more generally.

## Model

The implementations of the both the rule-route (RULEX) and exemplar-route (EBRW) relied on the mechanisms within the ACT-R declarative memory module (see Anderson et al., 2004 for details). In ACT-R, declarative knowledge is represented in the form of chunks. Chunks were used to represent both the explicit categorization rule used by the rule-route and the rule-compliant training exemplars that were used by the exemplar-route. We shall first describe in more detail how these two routes in the model were implemented before describing how the model choose between routes when making categorization decisions.

A central component of the model is that both routes rely on the retrieval of chunks from declarative memory. The rule-route in the model determined category membership by first attempting to retrieve a chunk representing the explicit categorization rule. If this was retrieved, the test items were compared to the rule and if they matched all of the rule relevant features a positive response was given; if a feature did not match the rule specification then a negative response was given. As will be explained in detail below, rule retrieval occasionally failed because the chunk’s activation was below the retrieval threshold. On these rare occasions the model made a random (guessing) response.

The exemplar-route in the model made response judgments by attempting to retrieve chunks representing rule-compliant exemplars (which were studied during training). If an exemplar was successfully retrieved, then the model made a positive response (because all training items were compliant with the rule). If a training exemplar could not be retrieved (because the activation of the chunks where all below the retrieval threshold), then a negative response was made, indicating that the test item was not a member of the category.

The main difference between these two routes then is that rule-route uses only the rule-relevant features to cue the retrieval of the rule from declarative memory, whereas the exemplar-route uses all of the features of the test item to cue the retrieval of past training exemplars from declarative memory. In other words, the rule-route of this model is functionally equivalent to an exemplar model that gives weighted attention *only* to the diagnostically relevant stimuli features.

The above sets out the core ideas for how the two routes in the model work. To give a more detailed understanding, we next expand on the mechanisms of the ACT-R declarative memory module used by the model.

As outlined above, each route relied on the retrieval of chunks from declarative memory. The activation of a chunk *i* (*A_i_*) is defined as

$$A_{i}=B_{i}+\underset{l}{\Sigma }{PM}_{li}+\epsilon \left(\text{activation equation}\right)$$

where, *B_i_* is the base-level activation of the chunk *i*, *PM_li_* is a partial matching score that computes the similarity of chunk *i* to the current test item *l* (which is vital for the exemplar-route), and ε adds noise to the system.

The base-level activation gives a temporary boost in activation to a chunk after it is retrieved from memory. This reflects the general idea that chunks used recently and frequently are more likely to be needed again in the future and the rate of base-level learning of a chunk is based on the rational analysis of Anderson and Schooler (1991). It is given by the equation

$$B_{i}=In(\overset{n}{\underset{j=1}{\Sigma }}t_{j}^{-d})\left(\text{base-level learning equation}\right)$$

where, *n* is the number of presentations for chunk *i*, *t* is the time since the *j*th presentation, and *d* is a decay parameter (which is set to the default value of 0.5, as a value that has emerged as appropriate over many applications, see Anderson et al., 2004). This means that over successive trials chunks that are used often have their activation strengthened allowing them to be retrieved more rapidly on future trials. This is particularly pertinent to the rule-route in the model: there is only a single chunk representing the rule, and so its activation is strengthened over consecutive trials in which it is used. The same also applies to the exemplar route but to a lesser extent since there are more exemplars in memory that could be potentially retrieved. How then does the model decide which exemplar to retrieve?

The partial matching component of the activation equation is critical to the exemplar-route as it allows for gradation in exemplar similarity between a test item and the training exemplars stored in declarative memory. Without it, the difference between high- and low- similarity items cannot be captured. The matching score in the activation equation is a sum computed over all of the dimensions of the retrieval request from the test item. The match scale *P* reflects the weight given to a dimension, and this was set to the default value of 1 (we discuss the implications of differential weighting in detail below). The match similarities *M_li_* determine the similarity between the feature in the retrieval specification and the corresponding dimension of the exemplars in memory. In the reported simulations, each match increased activation^1^ by 1 and each mismatch reduced activation by -1. To give an example, the stimuli used in Hahn et al. (2010) were defined along six feature dimensions. Suppose a test item matched on four of the six features with a training exemplar, the partial matching score would therefore be 2 (i.e., 4 - 2). In this way, high similarity test items, which by design had more features overlapping with the training exemplars, would have higher match scores than low similarity test items, which shared fewer features with the training exemplars.

Finally, we consider how activation values determine the likelihood and duration of chunk retrievals from declarative memory. In ACT-R, the probability that the activation of chunk is greater than a threshold τ is given by the following equation:

$$P_{i}=\frac{1}{1+e^{-(A_{i}-\tau )/s}}\left(\text{probability of retrieval equation}\right)$$

where *s* controls the noise in the activation levels. In the reported simulations the retrieval threshold τ was set to 0 (ACT-R default value). If a chunk is successfully retrieved, the activation of the chunk also determines how quickly it is retrieved. The time to retrieve the chunk is given as

$$T_{i}={Fe}^{-A_{i}}\left(\text{latency of retrieval equation}\right)$$

where *F* is a latency factor, which was set to 1 (ACT-R default value).

### Choosing between Routes

We next describe how the model decided which route to use when making a categorization decision using the ACT-R procedural memory system. The Anderson and Betz (2001) model assumes that categorization judgments are made on a trial-by-trial basis by choosing the route–either rule-route or exemplar-route–that has the greatest expected utility. Anderson and Betz define utility as a trade-off function between the probability *P* that the route would be expected to lead to a correct judgment and the expected time cost *C* required to reach that judgment, such that the utility *U* of route *i* is,

$$U_{i}=P_{i}G-C_{i}+\epsilon \left(\text{production utility equation}\right)$$

where *G* is a constant that reflects the value of the objective (which can be thought of as a maximum time investment to achieve the goal) and ε represents a noise parameter.

The route with the greatest utility is used to make a categorization judgment on a trial-by-trial basis. However, because utility estimates are noisy, over many trials the aggregate output of the model will reflect a blending of the rule-route and exemplar-route responses. But as we shall show in the next section, it is also possible to systematically vary the utility values of each route so as to bias the model to favor one route over the other (by changing the probability *P* that the route would be expected to lead to a correct judgment).

A further benefit of the ACT-R framework is that it gives the modeler the choice to either use constant utility values or use dynamic utility values that are updated based on experience (i.e., utility learning). As we shall explain in more detail below, we use this utility learning mechanism to evaluate a supervised version of the model. In this supervised version of the model, where utility learning is enabled, the estimated utility of a route is updated after every time it is used. This is done by updating the probability *P* that the route is expected to lead to a correct judgment:

$$P=\frac{Successes}{\left(Successes+Failures\right)}\left(\text{probability of success equation}\right)$$

where *successes* is a count representing the frequency of positively rewarded responses attributed to the route (i.e., correct responses) and *failures* is a count of negatively rewarded responses (i.e., error responses). In the unsupervised version of the model this utility learning mechanism was disabled.

## Modeling Experiment

**FIGURE 1:**
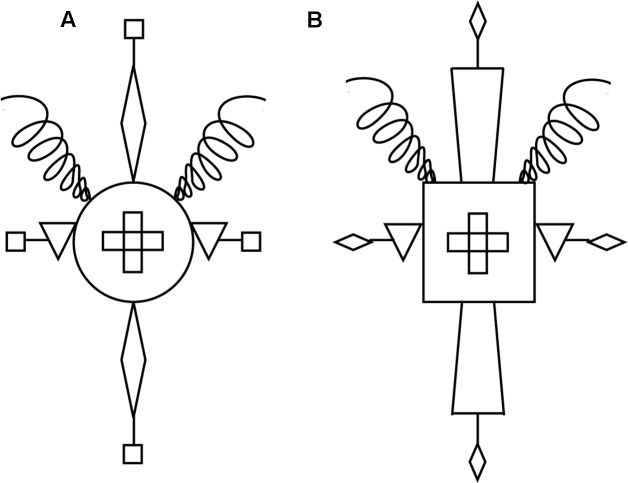
**Examples of the material used in Hahn et al. (2010).** Example **(A)** is a high-similarity rule compliant item and example **(B)** is a low-similarity compliant item. The rule states that an object “is an A if it has an upside-down triangle at the sides, a cross in the center, and a curly line at the top.”

We evaluate both a supervised and an unsupervised version of the model against data from Hahn et al.’s (2010) study. In these studies, participants were first informed of the relevant rule for category membership: the simple rule used in the two studies that we model specified three necessary and sufficient features for category membership (e.g., “is an A if it has an upside-down triangle at the sides, a cross in the center, and a curly line at the top”). After being told this rule, participants were shown 12 instances of rule-compliant items, three times each. **Figure 1** gives an illustration of two rule-compliant items from this experiment.

Analogously, the model represented the rule in declarative memory, and was presented with the same training items. These training exemplars were stored in declarative memory for later retrieval by the exemplar route. The model encoded all of the stimulus features, assuming an overall visual encoding time of 555 ms to encode each item.

After viewing the training items, the model, like the participants, was given 96 previously unseen test items to classify. Test items were presented over four blocks of 24 trials; we maintain this for the model to facilitate comparison. Following categorization, test items were not added to declarative memory. Thus, the exemplar-route relied entirely on the recall of items viewed at training (a modeling decision which we return to below).

As indicated above, Hahn et al.’s (2010) studies were designed such that the manipulation of similarity was orthogonal to category membership. Specifically, half of the test items complied with the rule, and half violated it. At the same time, half of the test items were high in similarity to the training exemplars that participants viewed at the start of the experiment, and half were low in similarity to these training exemplars. The degree of similarity between training and test items was determined by the number of irrelevant features that items had in common (i.e., features that were not mentioned in the rule). **Figure 1** gives an example of what these stimuli looked like, showing a high-similarity rule-compliant item (a) and a low-similarity compliant item (b). In Hahn et al.’s experiments, each test item was unique and different to the training exemplars. High-similarity test items had two rule irrelevant features (e.g., the Body and the Antenna of the stimuli) that matched the features seen in one third of the training exemplars. In the case of the example shown in **Figure 1A**, the Body is a circular shape and the Antenna extensions are squares. In contrast, low-similarity test items had values for the same rule irrelevant features that were never seen in the training exemplars. In the case of the example shown in **Figure 1B**, the Body is a square shape and the Antenna extensions are triangles. These shapes were never used in the training exemplars for these rule irrelevant features. (Please see Appendix of Hahn et al., for a complete logical description of all 12 training exemplars and 96 test items used in these experiments).

To evaluate the model, we systematically varied the utility of the exemplar-route so that it would be selected over the rule-route on 0–100% of trials (in increments of 10%). In other words, rather than letting the model learn route utilities we simply explored the range of possibilities directly. Exploring this aspect of the parameter space allowed us to quantify the relationship between the size of any response time difference between high- and low-similarity items and the presence of classification errors. Both error rates and response time differences are directly related to the core theoretical assumptions of the model, in that, given the nature of the test items, systematic errors arise *only* through the use of the exemplar route, as do the response time differences between high- and low-similarity exemplars. The rule has no exceptions, so always leads to correct classification, whereas, by design, exemplar similarity is orthogonal to category membership, so that exclusive use of the exemplar route would lead to chance performance of 50% accuracy. By the same token, the rule is blind to similarity (as it focusses only on the rule relevant features) so its use cannot give rise to exemplar similarity effects. As consequence, correcting an excessive number of total errors means that the exemplar route must have been used on proportionally fewer trials; however, reducing the relative usage of the exemplar route necessarily reduces any effect of similarity on reaction time data. These two aspects of the data might not be trivial to satisfy. In other words, it is an open question whether the model can be made to simultaneously fit the data along these two measures.

In our exploration of the parameter space, we first sought to fit an unsupervised version of the model to data from a version of the experiment in which participants did not receive feedback on the accuracy of their categorization judgments (Experiment 1 in Hahn et al., 2010). Following this, we compared the performance of a supervised version of the model to data from a version of the experiment in which participants received explicit feedback after each trial on the accuracy of their categorization judgment (Experiment 3 from Hahn et al.). To compare model and experimental results, we simulated a population of ‘model participants.’ This approach was necessary because the model’s behavior is stochastic, and multiple model runs were necessary to obtain reliable estimates of error rates. The model was run over the experimental procedure 50 times and performance averaged across independent runs.

## Results

### Unsupervised Model

**FIGURE 2:**
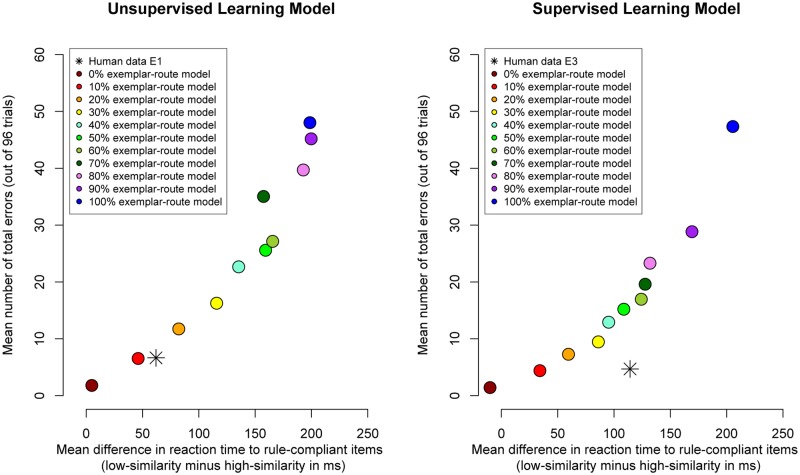
**Data plots of the difference in reaction time to rule-compliant items (the exemplar similarity effect) against number of classification errors.** Circular data points represent modeled data. Each circular data point shows the results of running the model with a different weighting of exemplar-route relative to rule-route (from 0 to 100% in increments of 10%). The star data points represent human data are from Hahn et al. (2010) Experiment 1 (left panel, unsupervised) and Experiment 3 (right panel, supervised). Note that in the human data the similarity effect on latencies increases from Experiment 1 to Experiment 3, while the error rate goes down.

The left panel of **Figure 2** plots the difference in reaction time for correct responses to high- and low-similarity compliant items against total number of classification errors. The figure shows the strategy space of the model: Each circular data point represents increasing use of the exemplar-route relative to the rule-route (from 0 to 100%, in increments of 10%). For example, the left-most data point in the figure (dark red circular data point) shows the model’s performance when the rule-route was used exclusively. The model made only two errors on average (across 98 trials) but there was also a very small difference in response times (response times were on average only 5-ms faster for high-similarity items than for low-similarity items). With increasing use of the exemplar-route (moving left-to-right through the circular data points in the figure), the difference in response times between high- and low-similarity items increased. Critically, this increase in exemplar-similarity effect was associated with an increase in response errors. For example, the right-most data point in the figure (blue circular data point) shows the model’s performance when it exclusively used the exemplar-route: many response errors were made (48 errors across 98 trials) but there was a relatively large difference in response times (response times were 199-ms faster for high-similarity items than for low-similarity items).

**Figure 2** also shows the human data from Hahn et al. (2010) Experiment 1 (star data point). Considering the overall number of errors heavily constrains the fit of the model to the human data because even a modest increase in the relative use of the exemplar-route is associated with an increase in classification errors. It can be seen in the figure that the model fits both the error rate and reaction time data from Experiment 1 in Hahn et al. when the exemplar-route is used on 10% of trials (red circular data point). When the model used the exemplar-route on 10% of trials it made very few response errors (7 errors across 98 trials, which is consistent with the error-rate found in Experiment 1 of Hahn et al.). There was also a difference in response times to rule compliant items (response times were 46-ms faster for high-similarity items than for low-similarity items, which is consistent with the 62-ms difference reported in Experiment 1 of Hahn et al.). These findings suggest that given a thorough sweep of the model’s parameter space (systematically exploring the relative use of exemplar-route over the rule-route), it is broadly possible to fit both the error-rate and reaction time data from Experiment 1 in Hahn et al.

**FIGURE 3:**
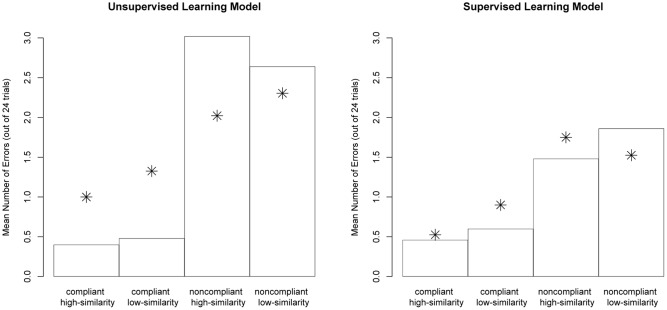
**Data plots of error-rates across experimental conditions.** Bars represent modeled data. The model was initially biased to use the exemplar-route on 10% of trials. The star data points represent the human data are from Hahn et al. (2010) Experiment 1 (left panel, unsupervised) and Experiment 3 (right panel, supervised).

We next report on a detailed assessment of the performance of the best fitting 10% exemplar-route model and compare that to the observed human data from Hahn et al. (2010). **Figure 3** (left panel) shows the distribution of errors found in Experiment 1 of Hahn et al. (represented in the figure by the star data points) and the distribution of errors made by the unsupervised 10% exemplar-route model (represented in the figure by the bars). The core data to capture are that participants made significantly fewer errors when categorizing high-similarity compliant items than low-similarity compliant items. The model fails to capture the effect of similarity on error-rates: There was an equal number of errors made for high- and low-similarity compliant items. In contrast, the model generated differences between the high- and low- similarity non-compliant items, whereas the human data contains no significant differences.

Finally, **Figure 2** makes clear that when reliance on the exemplar-route was increased the frequency of errors increased substantially relative to those observed in the human data. Fitting the number of errors therefore heavily constrained the model.

**FIGURE 4:**
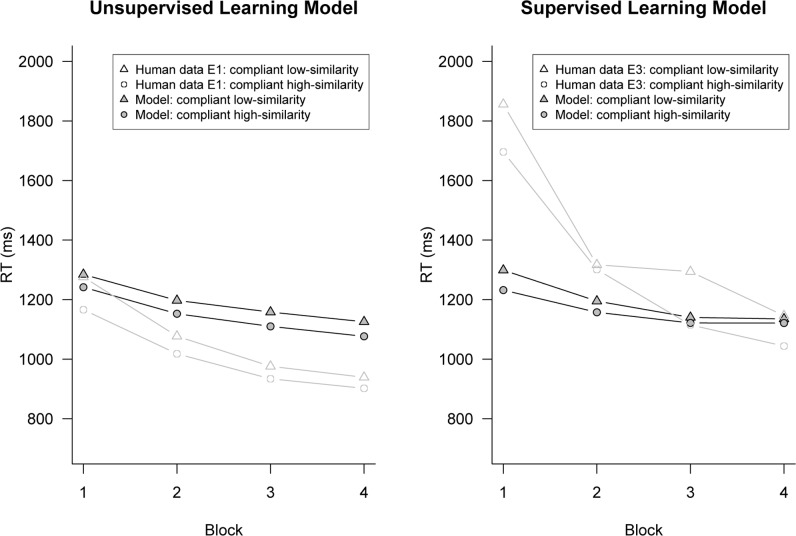
**Data plots of response times for high- and low-similarity rule-compliant across block.** The model was initially biased to use the exemplar-route on 10% of trials. Human data are from Hahn et al. (2010) Experiment 1 (left panel, unsupervised) and Experiment 3 (right panel, supervised).

We next consider how well the 10% exemplar-route model fit the absolute reaction time data. **Figure 4** shows the average response time for high- and low-similarity compliant items across each block of trials in the experiment. As expected, the model demonstrated a speed-up in reaction time over successive blocks of trials because of the strengthening of chunks in memory (i.e., the base-level learning equation). More importantly, like the human data, the exemplar-similarity effect predicted by the model did not diminish over time and was still present in the final block.

### Supervised Model

Experiment 3 in Hahn et al. (2010) used the same design and materials as the first study but additionally gave participants feedback at the end of each trial on whether their response had been correct or not. Despite giving this additional feedback, Hahn et al. found an exemplar-similarity effect on response times: Participants were again faster at categorizing high-similarity compliant items than low-similarity compliant items.

We ran the model through a supervised version of Hahn et al.’s experimental procedure. To do this we enabled ACT-R’s utility learning mechanism and gave the model explicit feedback on its categorization accuracy after each trial. As before, we systematically varied the *initial* utility of the exemplar-route so that it would be selected over the rule-route on 0–100% of trials (in increments of 10%). Note that a crucial difference here is that after each trial, the model updated the estimate of a route’s probability of success based on feedback (i.e., whether a correct or incorrect response had been made). This was done by using the probability of success equation (see above for details). Every correct response increased the probability of success estimate for a route, making it more likely to be selected on future trials. In contrast, every incorrect response decreased the probability of success estimate of a route, making it less likely to be selected on future trials.

The right panel of **Figure 2** shows for the supervised version of the model the difference in reaction times for high- and low-similarity items against classification errors. As before, each model data point represents an increase in the initial use of the exemplar-route relative to the rule-route. Comparing these two panels, for almost every parameter value of the model, the supervised model (right panel) made fewer classification errors than the unsupervised model (left panel). For example, considering the 50% exemplar-route model (green circle), we see that the supervised model made 15 errors whereas the unsupervised model made 26 errors. Both models started out identical; only the supervised model learnt over time to avoid the error-prone exemplar-route and so made fewer errors. This strategic disfavoring of the exemplar-route necessarily diminished the size of the exemplar similarity effect in the response time data. For example, the 50% exemplar-model showed a 135-ms exemplar similarity effect when run as an unsupervised model. When the same model was run as a supervised model, the exemplar similarity effects decreased to 95-ms. This general pattern is seen across the parameter space of the model (with the obvious exception of the 100% exemplar-route model).

We next consider whether the supervised model can fit both the reaction time and error-rate data from Experiment 3 of Hahn et al. (2010). It can be seen in the right panel of **Figure 2** that across the parameter space of the model (i.e., varying the initial use of the exemplar-route relative to the rule-route), the model cannot fit both the observed reaction time and error-rate data from Experiment 3 of Hahn et al. (star data point in right panel of **Figure 2**).

In terms of error-rate data, the 10% exemplar-route model (red circle) provided a good fit to the human data: errors were made on four trials (out of 96) for both model and data. Interestingly, the unsupervised 10% exemplar-route model also provided the best fit to the data from Experiment 1 of Hahn et al. (2010). Because of feedback, the supervised model made fewer errors than the unsupervised model, and this was consistent with the observed differences between Experiments 1 and 3 of Hahn et al.

While the supervised model fit the error data well, there was a poor fit to the reaction time data: the model showed a difference of only 34-ms between high- and low-similarity items, whereas the data from Experiment 3 of Hahn et al. (2010) showed a 114-ms difference. That is, the model seems to under-predict the size of the exemplar similarity effect on reaction time given the observed low rate of classification errors. To understand why this is, **Figure 4** shows the average response time for high- and low-similarity compliant items across each block of trials. For the model, the difference in reaction times between high- and low-similarity items decreased over each block of trials. This is because the model was learning with experience to avoid the error-prone exemplar-route, and this diminished the exemplar similarity effect. In contrast, the data from Experiment 3 of Hahn et al. shows a robust exemplar similarity effects, even in the last block of trials, and no evidence for block by similarity interactions. This points to a fundamental failure of the model to fit the data.

## Modeling Alternatives

In any modeling enterprise, choices must be made. Before moving on to the general implications of the results presented, we examine some of the modeling assumptions that we made and the extent to which they may be crucial to our results. Three assumptions warrant closer scrutiny: the parameters used to calculate match scores in the model, the decision not to store classified test items in memory, and the allocation of attention to the different stimulus dimensions.

### Match Scoring

We noted above that when calculating partial match scores the ACT-R default value for matches is zero. Partial matching is usually used to penalize the activation of items that do not fully match the retrieval request; in contrast, by using a match score of 1 we give an activation boost for matches. Similarity values are a parameter of the model that can be set by the modeler along with the scale on which they are defined. The Anderson and Betz (2001) implementation of the EBRW used the default which amounts to the city block metric as a measure of distance. This is a departure from Nosofsky and Palmeri’s (1997) original EBRW which used a Euclidean distance, with distances converted to similarities via an exponential decay function as standard in Nosofky’s Generalized Context Model (Nosofsky, 1986; Nosofsky, 1988). The immediate question of interest is the extent to which such differences in choice of similarity measure will impact model performance. As Anderson and Betz demonstrate, their use of the city block metric does not prevent them from fitting Nosofsky and Palmeri’s (1997, Experiment 1) data; and our metric is a simple linear transformation of Anderson and Betz’s city block metric^2^. More fundamentally, however, the extent to which a change of similarity metric can alter model performance depends on the nature of the items set. The item set of Hahn et al. (2010) is carefully balanced by design. Recall that the critical comparisons are between high- and low- similarity test items, where ‘high’ and ‘low’ similarity are defined relative to the training items. By design, each of the test items in the high similarity and low similarity categories, respectively, has the same number of matching/mismatching features to all items in the training set as every other test item in its condition (see Appendix). Any monotonic transform of this space, whether it be linear or non-linear (such as Nosofsky and Palmeri’s metric), will simply serve to scale the differences between test items without changing their individual rank order. In other words, Nosofsky and Palmeri’s metric will accentuate the difference between the closest and the further distances due to the exponential decay function, enhancing the difference between high similarity and low similarity test items. Change of metric will accentuate or diminish the magnitude of the similarity effect in terms of latency differences. However, the ultimate failure of the model does not lie with its inability to produce appropriate latency differences, nor with its ability to do so while matching the overall error rates seen in the human data: it does both of those things just fine. What the model falls down on is fundamental qualitative properties: (1) it generates a difference in errors on high- and low-similarity items for non-compliant items, but this similarity effect is not generated for compliant items – which is the opposite of the human behavior; (2) the similarity effect diminishes by necessity once feedback is introduced; and (3) the latency difference in the human data is bigger in the feedback condition even though the errors are lower, a qualitative shift the model cannot generate (at least using the same parameters across both tasks). These problems arise from the interaction of the fundamental design characteristic of the model, namely that route selection is governed by route utility, and the nature of the specific stimulus set in which exemplar similarity is entirely orthogonal to category membership. The details of the scale factor applied by a specific similarity metric are irrelevant to this problem.

### Exemplar Storage

We also assumed that only training exemplars were stored and used for forming classification judgments. However, many exemplar theories (e.g., Logan, 1988; Nosofsky and Palmeri, 1997) assume that exemplars are stored for every classification decision that is made. Therefore, a reasonable alternative might have been to add successive test items to declarative memory once decisions on them had been made. Although we did not evaluate an alternative ACT-R model that incrementally added test items to memory, we did, nevertheless explore the likely impact that adding exemplar representations of classified test items to declarative memory would have had for our pattern of modeling results. Specifically, we conducted an analysis that compared the mean similarity of test items for each condition across the entire set of test items with that across the set of training examples. The resultant values characterize the (asymptotic) information present within the test items were these to be classified accurately.

**FIGURE 5:**
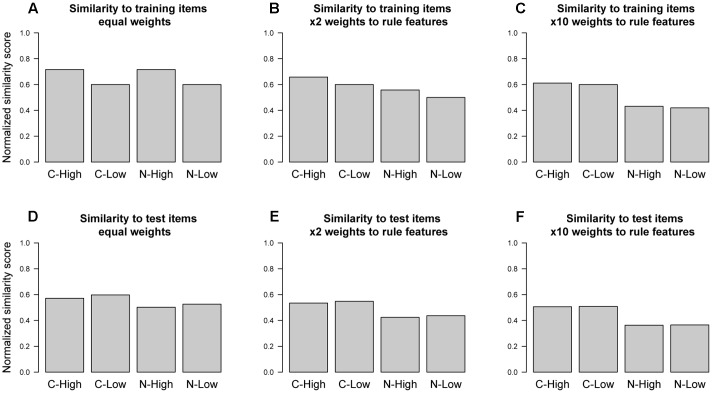
**Mean similarity of test items for each condition to **(A–C)** training items and **(D–F)** test items across varying weight allocations to stimulus features**.

**Figure 5** shows the normalized mean similarity scores for each experimental condition across the set of training examples and the set of test items. As expected, across the training examples (**Figure 5A**) there is a difference in similarity between the high- and low-similarity conditions, regardless of rule compliance (i.e., compliant high-similarity and non-compliant high-similarity have greater similarity score than compliant low-similarity and non-compliant low-similarity). By design, the test items factorially combined rule compliance and non-compliance with high- and low-similarity to the initial training exemplars, with each category present in equal number. It is consequently no surprise that if we calculate similarity scores by comparing individual test items to the entire set of test items (**Figure 5D**), there is no such difference between the high- and low-similarity conditions. Therefore, adding the test set in entirety does not, on average, alter the similarities set up by the training items. Hence, even if the model stored exemplar representations of the classified test items, this would not systematically alter the effect of paying attention to similarity.

There are further concerns to the idea of adding test items to memory. One of the main barriers is that a categorization label would need to be associated with each test item in memory. In the unsupervised version of the task (Experiment 1 of Hahn et al., 2010) the only way for the model to label an item would be based on its own category decision. However, as **Figure 2** clearly shows the model makes many classification errors when using the exemplar-route because of the way that category membership was designed to be orthogonal to similarity; the above analysis shows that this persists even when test items are added. As a result, the assigned category label would be incorrect most of the time–many items that were not category members would be incorrectly added to memory as positive instances of the category. This in turn would lead to the model making even more classification errors when using the exemplar-route, and so failing to fit the human data.

A final concern reflects the nature of the ACT-R activation equation. Even if training items had been added to memory, they would unlikely have been used. This is because at the start of the experiment only the training exemplars would be in memory. Each time one of these training exemplars is retrieved from memory it would receive a boost in base-level activation, reflecting the recency and frequency of usage. Critically, this base-level activation would likely operate to make these training exemplars dominate over the less frequently used test items.

### Feature Weighting

A broader concern with generalizing these results to other exemplar-based models is that the implementation of Nosofsky and Palmeri’s (1997) EBRW model taken here assumes that attention is equally distributed between stimuli features when forming a classification judgment. This assumption of equal weights is not commonly held across exemplar theories (e.g., Nosofsky, 1988, 1992; Kruschke, 1993), which instead allow for greater weight to be given to diagnostically relevant stimuli features. As mentioned above, an exemplar model with optimal attention weights would be identical, in terms of classification behavior, to the rule – this is because the features that are specified in the rule are the only features that are important for the classification task and so are the features that would come to be weighted more saliently.

It might still be the case though that there are other non-optimal sets of weights, other than equal weighting, that might give rise to better fits between model and data. To explore this issue, we conducted analyses of the effect of differential feature weighting on mean similarity.

**Figure 5** indicates the way additional weight to the rule-relevant features changes similarity measures across conditions. We consider first the standard case where only training items are included as reference points. As expected given the design of the materials, **Figure 5A** shows that when all feature dimensions are given equal weighting, the high-similarity test items (compliant high-similarity and non-compliant high-similarity) are more similar to the training items than the low similarity test items (compliant low-similarity and non-compliant low-similarity). Because these similarity manipulations are orthogonal to rule membership, judgments formed based on exemplar similarity to training items would be at chance. In contrast, if a match along a rule-relevant feature is heavily weighted (i.e., given ×10 the weight, as in **Figure 5C**), then the difference between items in the high-similarity (compliant high-similarity and non-compliant high-similarity) and low-similarity (compliant low-similarity and non-compliant low-similarity) conditions disappears because exemplars now approximate the rule. Values in between these two extreme points give rise to blends of both properties. For instance, **Figure 5B** gives twice the weight to rule features as to non-rule features. While this leads to a small difference between items in the high-similarity (compliant high-similarity and non-compliant high-similarity) and low-similarity (compliant low-similarity and non-compliant low-similarity) conditions, it also provides some information about category membership. Such an intermediate blend, in effect, mirrors the aggregate behavior of our hybrid ACT-R model that sometimes uses the rule route (i.e., that uses only the rule-relevant features) and sometimes uses the exemplar route (i.e., that gives each feature equal weight). In other words, the size of any exemplar-similarity effect in this task is necessarily associated with classification errors, even when differential feature weights are allowed.

The same conclusion, finally, emerges from consideration of the resultant similarities if all items, not just the training items, are considered. Consideration of **Figures 5D–F** reveals that differential weights have no benefit whatsoever here, as the essential difference between high- and low-similarity conditions is never observed. This further supports the above contention that nothing would be gained by incrementally adding test items to declarative memory.

In summary, we can therefore be confident that our results do not rest on the particular choices that were made in our modeling work about exemplar storage and exemplar weighting. At the heart of the model’s difficulties lies the fact that attention to similarity is detrimental to categorization accuracy by virtue of stimulus design. A focus on accuracy necessarily drives down attention to non-rule features, whether through use of the rule route or through feature weighting, which in turn means the similarity effects will go away, with the model failing to fit the data as a result. The features on which the similarity manipulation rests are irrelevant by design, neither differential weighting nor adding exemplars to memory can alter this.

### Other Models

Although we have chosen to focus on the Anderson and Betz (2001) categorization model, it is worth considering other prominent categorization models in the literature and highlighting some of the challenges that they may face in accounting for the pattern of results found by Hahn et al. (2010).

The dataset described here would seem to be equally problematic for the model of Ashby et al. (1998, see also, Ashby and Ell, 2002; Ashby and Casale, 2005). This model conflicts with the results of Hahn et al.’s (2010) two experiments both because of its route interaction and because the exemplar similarity effects that Hahn et al. observe cannot readily be recast in the decision-bound framework of the model.

Ashby et al.’s (1998) model assumes the existence of two fundamentally distinct systems on which classification can be based. One of these is an implicit (procedural) system, the other is a system based on verbal (or verbalizable) rules. As the model’s name COVIS (competition between verbal and implicit systems) indicates, the two systems are assumed to be in competition, with initial dominance of the verbal system. The concept of verbal rule is operationalized roughly as “any strategy that can be described verbally” (Ashby et al., 1998, p. 445). In the model, such rules are translated into decision-bounds. The explicit, experimenter-provided rules of Hahn et al. (2010) are not just verbalizable, but can also be readily translated into a decision boundary in the multi-dimensional space of their stimulus materials, as we demonstrate shortly.

The first problem for the COVIS model, as defined by Ashby et al. (1998), is that the similarity effect observed in Hahn et al. (2010), which must be mediated by the implicit system, should never arise because the rule is perfectly predictive. As Ashby et al. (1998, pp. 458) state: “A fundamental assumption of COVIS is that in learning of some new category structure, there is a large initial bias in favor of the verbal system. The implicit system can overcome this bias only if it is reinforced at a higher rate than the verbal system over some reasonably long number of trials.” However, there is no reinforcement for the implicit system, because the rule in all experiments is entirely predictive. The implicit system should thus not become operative in the context of Hahn et al.’s Experiments 1 and 3, and hence the similarity effects are left unexplained.

Furthermore, the nature of the implicit system as proposed by Ashby et al. (1998) seems unable to account for the specific similarity effects that Hahn et al. (2010) observe. In characterizing the nature of the implicit component of their model, Ashby et al. (1998, p. 445) considered both decision-bound and exemplar-theory as possible alternatives, but decided in favor of a decision-bound approach.

In the Hahn et al. (2010) experiments that have been the focus of the modeling efforts in the present paper, test items that were high in similarity to the initial training examples were responded to more quickly and more accurately. In order for a decision-bound model to explain this effect, the difference between high- and low-similarity items must translate into respective differences in distance from the decision-bound. This idea has been developed in previous debate between decision-bound and exemplar theorists in the wider categorization literature in order to explain (non-rule-based) studies which found that exemplars high in similarity to other exemplars are responded to more accurately and quickly (e.g., Ashby et al., 1994; Lamberts, 1995, 2000; Nosofsky and Palmeri, 1997). One explanation of these findings assumes responses are faster because of similarity to previous exemplars (Lamberts, 1995, 2000; Nosofsky and Palmeri, 1997). By contrast, decision-bound theory, which eschews the representation of individual exemplars, has claimed that it is distance from the decision-bound that is responsible for these effects (see, Ashby et al., 1994): high typicality items can be responded to more quickly because they are farther from the category’s decision-bound. The studies in question have been inconclusive with regards to this issue, because exemplar similarity and distance from the decision-bound have correlated. However, by design these two aspects are dissociated in Hahn et al.’s materials.

**FIGURE 6:**
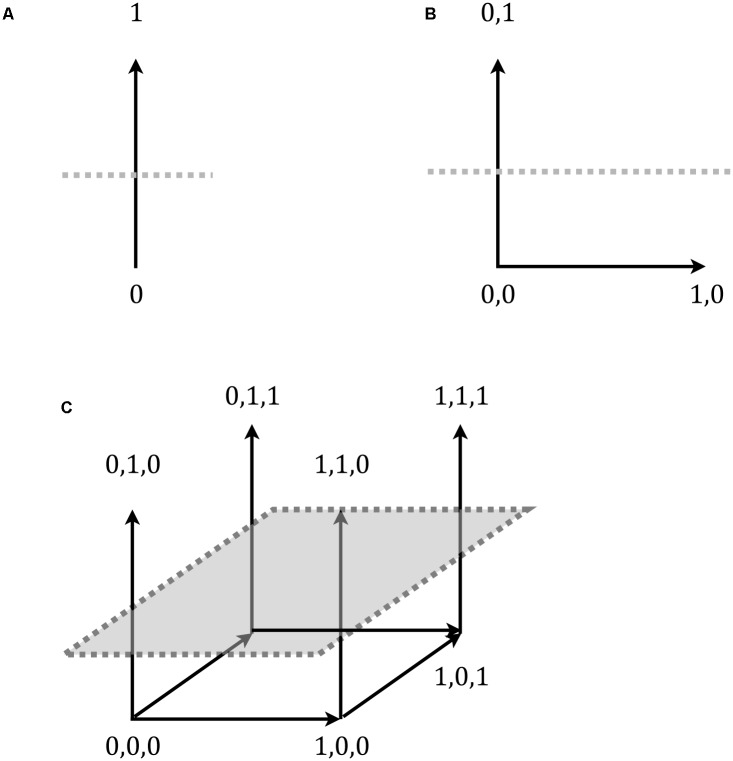
**Decision-bound (dotted line) in a category space of binary-valued materials** **(A)**. The corners correspond to the possible exemplars. As can be seen, adding in additional, irrelevant, non-rule dimensions in **(B,C)** does not alter the distance to the decision-bound. However, the irrelevant dimensions can be used for manipulating similarity. Of the four corners above the decision-bound in **(C)**, item 0,1,0 is less similar to item 1,1,1 than are the remaining two items, with degree of similarity represented by gray scale in the illustration.

Hahn et al.’s (2010) high- and low-similarity test items differ in similarity to the training exemplars, but they do *not* differ in their distance from the category’s decision-bound. As noted above, the simple rule used in the two studies that we model here specified three necessary and sufficient features for category membership. Namely, an item was a category member if “it has an upside-down triangle at the sides, a cross in the center, and a curly line at the top.” **Figure 1** provides an illustration of the accompanying items, showing both a high-similarity rule-compliant item (a) and a low similarity compliant item (b). As can be seen the materials were not based on differences along continuous valued dimensions, as in previous contrasts between exemplar- and decision-bound models, but rather on discrete, substitutive features (on this contrast more generally see e.g., Tversky, 1977); most importantly, however, the rule makes reference to the values of only some of these features. Why this decouples exemplar similarity and distance from the decision-bound is illustrated in **Figure 6**.

**Figure 6A** shows a single dimension of variation, where, for example, a value of 1 corresponds to a triangle at the side, and a value of 0 represents a circle. A rule for category membership that says things are A’s only if they have a triangle at the side (i.e., a value of 1) corresponds to a decision-bound like the horizontal line. This bound separates the members of the category, items above the line, from the non-members below. As can be seen from **Figures 6B,C**, adding in additional rule-irrelevant dimensions (which would correspond to additional parts, or other attributes such as color) does *not* alter the location of this decision-bound. However, given enough additional, irrelevant dimensions, a similarity manipulation that is orthogonal to the decision-bound (i.e., the rule) becomes possible. For instance, in **Figure 6C**, one might present item 1,1,1 as a training exemplar. The further items “0,1,0,” “0,1,1,” and “1,1,0” could then be presented as novel test items. All are members of the category (they are above the decision-bound) and all are equally distant from the decision-bound. However, they are not equally distant, and with that similar, to *each other*. The item “0,1,0” is less similar to the training item (item “1,1,1”) than are the other two (items “0,1,1” and “1,1,0”), because it shares fewer dimension values (i.e., there is only one common value as opposed to two).

In Hahn et al.’s (2010) materials, the similarity relations among the rule compliant items are likewise based entirely on the rule-irrelevant dimension, which leaves distance to the decision-bound unaltered. Hence the observed increase in reaction times for the high-similarity items cannot be explained in terms of distance from the decision-bound. This also means that our results are evidence against decision-bound models of categorization in general (e.g., Ashby et al., 1994; see also Nosofsky, 1991; Nosofsky and Palmeri, 1997 for further evidence in form of typicality and frequency effects), not just COVIS with its decision-bound component.

Of course, it is typically possible for models and theories to accommodate conflicting results through further, additional assumptions; however, such assumptions must have some independent justification. One possibility suggested to us was that decision-bound models could readily accommodate our results by assuming that there is more noise in processing the feature instantiations of the low similarity items. However, some further explanation seems required, both as to why noise would have just the right effect on reaction times, accuracy and ratings, and as to why there should be more noise associated with these features in the first place. Because the features themselves are unrelated to the decision-bound, it is entirely unclear how even systematic noise would lead to the observed range of systematic effects on classification decisions. Given that the only characteristic of these features is that they are not those of the initial exemplars, it seems likely that alternative explanations will simply be introducing exemplars through the back door.

In summary, to accommodate our results, COVIS would seem to need to adopt an exemplar component and provide a different specification of how it interacts with the explicit, verbal route.

Finally, there are a few other hybrid models in the literature that are worth mentioning. The most prominent of these is Erickson and Kruschke’s (1998) ATRIUM model. It is a connectionist implementation of a rule module and an exemplar module, which is based directly on Kruschke’s (1992) ALCOVE, whose outputs are combined into an overall response via a gating mechanism that weights the predictions of each module. The materials used in Hahn et al.’s (2010) study present a different challenge to this model. The model is designed for categorization tasks involving so-called contrastive, or complementary, categories. In such tasks, there are two competing categories, say A and B, such that anything that is not an A is a B and vice versa, and the task is to learn to distinguish the two. Hahn et al., however, used a different category structure in that the three-feature rule highlighted above could be violated in many ways. Non-members simply fail to possess one of the rule-prescribed features, but there are many alternative ways in which each individual rule requirement can be broken, and these can, of course, also be combined. There is thus not a coherent alternative category in Hahn et al.’s materials other than whether the test items conform or not to the explicit rule. This structure was chosen because real world rules typically do not involve contrastive categories. For example, the criminal code contains a rule that forbids the offense of theft. The rule specifies theft through a positive characterization or description of what constitutes it. By contrast, the behaviors that are not theft are not delimited and, consequently are not given (and do not need) any specification. Moreover, such a non-contrastive structure has the methodological advantage of avoiding potential ambiguities about whether participants are searching for A features or B features in determining their response (see also Nosofsky et al., 1989). In its present form, ATRIUM has competing rule nodes within its rule component. It would thus require architectural modification in order to be at all applicable to the category structures used in the design of Experiments 1 and 3 of Hahn et al.

Even if we set aside the basic issue that ATRIUM requires contrastive categories, it is also unclear how and why a gating mechanism such as that specified in Erickson and Kruschke’s model would allow the model to factor in exemplar information. The rule route is itself entirely predictive, whereas the exemplar-route is not, and in the data from Experiment 3 of Hahn et al. (2010), participants receive constant feedback to that effect. It is hence unclear how a model based entirely around error correction could come to predict the sustained exemplar effects observed in Hahn et al.’s dataset. The same limitation, we believe, also applies to other connectionist hybrid models in the literature (e.g., Noelle and Cottrell, 1996, 2000).

Beyond hybrid models of categorization, finally, it may be tempting to assume that there are other general modeling frameworks that provide a ready-made solution to modeling these data. Drift diffusion models (DDM), for example, provide a general, and in many cases optimal, framework for two alternative forced choice tasks (see e.g., Bogacz et al., 2006). The binary classification decision faced by participants in the Hahn et al. (2010) task which involves a ‘yes’/‘no’ response on category membership would seem to fit that classification. In fact, the EBRW’s implements the discrete equivalent of a drift diffusion model (see Bogacz et al., 2006). This, in effect, answers the question concerning the applicability of DDM models to this task: the EBRW can fit the data reported here no more than the hybrid model containing it as a sub-component can. Exemplar similarity is non-diagnostic in this task and the ‘evidence’ – exemplar similarity – relies on rule irrelevant features, which by definition, are irrelevant to the task. This fundamental design feature of the Hahn et al. test set is what provides robust evidence for non-optimality in the resultant categorization decisions of participants. By the same token this design feature scuppers any model that will provide optimal category decisions at the present level of task reconstruction: the optimal behavior given a perfectly predictive rule is simply to use that perfectly predictive rule.

This leaves open the fundamental question of why human categorization is not optimal in this way, even though such optimality has been widely assumed. Answering this question will likely require a deeper understanding of visual attention, as Hahn et al. (2010) note. There are results within the literature on visual attention that involve exemplar effects. These studies suggest intriguing parallels for further examination. Specifically, these studies involve exemplar effects that seem relevant to the task of search for category relevant features in the first place. The first set of studies of interest stems from Chun and Jiang (1998, 1999) ‘contextual cuing’ paradigm. Directly relevant to the Hahn et al. task examined here is Experiment 1 of Chun and Jiang (1999). In this task, participants were presented with a visual search task that involved entirely novel objects as target and distractors. Participants either always saw the target object paired with the same distractors or paired with different displays of distractors, with overall exposure to a given ensemble of distractors held constant. Participants were faster with the consistent pairings, suggesting that the distractors primed the identity of the target object. Chun and Jiang (1998) also found evidence to suggest that target location could be primed in the same way. Interestingly, explicit memory regarding the target and distractor-display pairings was entirely at chance. Participants could not reliably distinguish between old and new contexts for a target. In other words, exemplar effects may be integral to categorization, because they are integral to establishing the presence or absence of the component features of the to-be-classified items.

An entirely different second study conducted by DeSchepper and Treisman (1996) supports this point. These authors found a negative priming effect for the unattended of two overlapping shapes after a single exposure. This negative priming effect could last, without decrement, across 200 intervening trials and across delays up to a month. Again, participants had no explicit memory of these shapes and were at chance in recognition, despite the robust priming effects, again suggesting a dissociation of implicit and explicit memory (see also Shacter et al., 1990). In other words, we suspect that exemplar similarity plays the role it does in the Hahn et al. (2010) experiments because exemplars are relevant to feature detection itself. By the same token, what has no utility at the level of classification in the Hahn et al. studies may have utility at the level of feature detection.

Hence, we think that what the model failures demonstrated in the present paper ultimately show, is that categorization behavior cannot really be understood without more detailed engagement with visual attention and feature recognition.

## Conclusion

It is hard to imagine a more appropriate task on which to test hybrid models of categorization than that used in Hahn et al. (2010) given that participants receive perfectly predictive, simple classification rules at the start of the task. The low error rates observed by Hahn et al. suggest these rules were successfully used by participants to categorize novel test items. At the same time, their data also show clear effects of an independent exemplar-similarity manipulation: Participants were faster at classifying novel test items that shared features, which were irrelevant to the categorization task, with items that were seen during an initial training stage in the experiment. Nevertheless, the hybrid model tested here, which combines Nosofsky et al. (1989) EBRW and RULEX models within the ACT-R cognitive architecture struggled to capture the behavioral data. Exploration of the model’s parameter space revealed that it was possible to generate very good quantitative fits for both the error rate and reaction time data from an unsupervised version of Hahn et al.’s task. But providing feedback about the accuracy of categorization judgments in a supervised version of the task revealed a critical weakness of the model because it predicted that the size of the exemplar-similarity effect should diminish over time as feedback demonstrates that paying attention to similarity is not a reliable cue to guide categorization judgments. The reason why the model predicts that the effect of instance-similarity diminishes over successive trials is quite clear: Exemplar-similarity effects are brought about through the use of the model’s exemplar-route. However, because the exemplar-route leads to frequent categorization errors, its utility is strategically lowered, which results in it being chosen less often. In contrast, the rule route, which does not give rise to any effect of instance-similarity, has its utility strategically increased because its use leads to correct judgments.

By the same token, all hybrid models we examined are challenged by these data because all of them assume that the relative influence of rule and exemplar processes is influenced by their utility. Moreover, the difficulties are compounded in the remaining models we examined because utility in these models would seem to reduce entirely to classification accuracy.

The idea of hybrid models remains attractive in principle (see also Hahn and Chater, 1998), but they do not yet seem sufficiently well-matched to actual behavior in practice. At the same time, there is evidence from a neural level for separable rule- and similarity-based processes in both categorization (see e.g., Folstein and van Petten, 2004; Koenig et al., 2005) and judgment (see recently e.g., von Helversen et al., 2014). However, exemplar similarity seems likely to be relevant not just to decisions about category membership itself, but also to the identification of category relevant features in the first place. Reconsideration of how and why rules and exemplars might computationally combine thus seems a pressing matter if human categorization behavior is to be fully understood.

## Author Contributions

All authors listed, have made substantial, direct and intellectual contribution to the work, and approved it for publication.

## Conflict of Interest Statement

The authors declare that the research was conducted in the absence of any commercial or financial relationships that could be construed as a potential conflict of interest.

## Footnotes

1 Partial matching in ACT-R is usually only used to penalize the activation of items that do not fully match the retrieval request so the default value is 0; in contrast, by using a match of 1 we give an activation boost for matches. We consider the implications of this parameter choice in the discussion below.

2 Specifically, our match function equals 2× the distance + the total number of features; that is, the possible values 0, -1, -2, -3, -4, -5, -6 are replaced with 3, 2, 1, 0, -1, -2, -3.
