# Supplementary material for: Ignore Similarity If You Can: A Computational Exploration of Exemplar Similarity Effects on Rule Application
Source: Front Psychol. 2017 Mar 21;8:424. doi: 10.3389/fpsyg.2017.00424 (PMC5359220; doi:10.3389/fpsyg.2017.00424)
Supplement: Supplementary file 1 [file Table_1.pdf]

Appendix for Distances of Test Items to Training Items

Brumby D and Hahn U (2017). Ignore Similarity If You Can: A Computational Exploration of Exemplar Similarity Effects on Rule Application. Frontiers in Psychology, 8:424. doi: 10.3389/fpsyg.2017.00424

Training items 1-12 are in columns, test items (1-24) in rows.

High Similarity Compliant

Distance to Training

Test Item	1	2	3	4	5	6	7	8	9	10	11	12 Training Item
1	1	2	2	2	2	3	3	3	2	3	3	3
2	2	1	2	2	3	2	3	3	3	2	3	3
3	2	2	1	2	3	3	2	3	3	3	2	3
4	2	2	2	1	3	3	3	2	3	3	3	2
5	1	2	2	2	2	3	3	3	2	3	3	3
6	2	1	2	2	3	2	3	3	3	2	3	3
7	2	2	1	2	3	3	2	3	3	3	2	3
8	2	2	2	1	3	3	3	2	3	3	3	2
9	2	3	3	3	1	2	2	2	2	3	3	3
10	3	2	3	3	2	1	2	2	3	2	3	3
11	3	3	2	3	2	2	1	2	3	3	2	3
12	3	3	3	2	2	2	2	1	3	3	3	2
13	2	3	3	3	1	2	2	2	2	3	3	3
14	3	2	3	3	2	1	2	2	3	2	3	3
15	3	3	2	3	2	2	1	2	3	3	2	3
16	3	3	3	2	2	2	2	1	3	3	3	2
17	2	3	3	3	3	2	3	3	1	2	2	2
18	3	2	3	3	3	2	3	3	2	1	2	2
19	3	3	2	3	3	3	2	3	2	2	1	2
20	3	3	3	2	3	3	3	2	2	2	2	1
21	2	3	3	3	3	2	3	3	1	2	2	2
22	3	2	3	3	3	2	3	3	2	1	2	2
23	3	3	2	3	3	3	2	3	2	2	1	2
24	3	3	3	2	3	3	3	2	2	2	2	1

Test Item

Low Similarity Compliant:

Distance to Training

Test Item	1	2	3	4	5	6	7	8	9	10	11	12 Training Item
1	3	3	3	3	3	3	3	3	3	3	3	3
2	3	3	3	3	3	3	3	3	3	3	3	3
3	3	3	3	3	3	3	3	3	3	3	3	3
4	3	3	3	3	3	3	3	3	3	3	3	3
5	3	3	3	3	3	3	3	3	3	3	3	3
6	3	3	3	3	3	3	3	3	3	3	3	3
7	3	3	3	3	3	3	3	3	3	3	3	3
8	3	3	3	3	3	3	3	3	3	3	3	3
9	3	3	3	3	3	3	3	3	3	3	3	3
10	3	3	3	3	3	3	3	3	3	3	3	3
11	3	3	3	3	3	3	3	3	3	3	3	3
12	3	3	3	3	3	3	3	3	3	3	3	3
13	3	3	3	3	3	3	3	3	3	3	3	3
14	3	3	3	3	3	3	3	3	3	3	3	3
15	3	3	3	3	3	3	3	3	3	3	3	3
16	3	3	3	3	3	3	3	3	3	3	3	3
17	3	3	3	3	3	3	3	3	3	3	3	3
18	3	3	3	3	3	3	3	3	3	3	3	3
19	3	3	3	3	3	3	3	3	3	3	3	3
20	3	3	3	3	3	3	3	3	3	3	3	3
21	3	3	3	3	3	3	3	3	3	3	3	3
22	3	3	3	3	3	3	3	3	3	3	3	3
23	3	3	3	3	3	3	3	3	3	3	3	3
24	3	3	3	3	3	3	3	3	3	3	3	3

Test Item
